# Supplementary material for: Single-Molecule Imaging of Wnt3A Protein Diffusion on Living Cell Membranes
Source: Biophys J. 2017 Dec 19;113(12):2762–7. doi: 10.1016/j.bpj.2017.08.060 (PMC5925569; doi:10.1016/j.bpj.2017.08.060)
Supplement: Document S1. Supporting Materials and Methods and Figs. S1–S6 [file mmc1.pdf]

Biophysical Journal, Volume *113*

Supplemental Information

Single-Molecule Imaging of Wnt3A Protein Diffusion on Living Cell Membranes

Anna Lippert, Agnieszka A. Janeczek, Alexandre Fürstenberg, Aleks Ponjavic, W.E. Moerner, Roel Nusse, Jill A. Helms, Nicholas D. Evans, and Steven F. Lee

Supplementary Material

Single-molecule imaging of Wnt3A protein diffusion on living cell membranes

---

Supplementary Figure 1. Photobleaching analysis.

Supplementary Figure 2: ATTO680-NHS labelling abolishes Wnt3A activity.

Simulation of single-particle diffusion

Parameters used for the data analysis using the Single-Dye tracking tool

Supplementary Figure 3: Localisation Precision of Data

Supplementary Figure 4: Freely diffusing Brownian motion of simulated Particles

Supplementary Figure 5: Wnt3A proteins stay longer associated with receptor expressing S2R+ cells than receptor free S2 cells.

Supplementary Figure 6: Continuous replenishment of diffusing Wnt3A proteins on cell membrane.

Supplementary Movie 1: Labelled Wnt3A Diffusing on S2 Cells with marked starting positions of the tracks.

Supplementary Movie 2: ATTO680-labelled BSA control on S2 cells.

Supplementary Movie 3: Heat inactivated Wnt3A does not diffuse on S2 cells.

Supplementary Movie 4: Labelled Wnt3A does not transfer across cell boundaries of adjacent S2 cells

Supplementary Movie 5: Simulated *vs.* Real Data

Supplementary Figure 1: Photobleaching Analysis

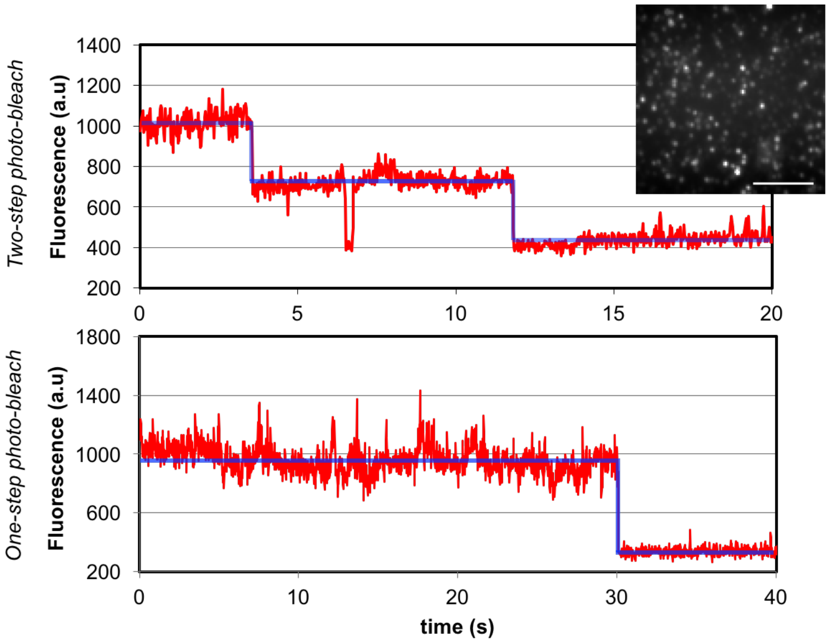

Supplementary Figure 1. Photobleaching analysis. Representative examples of integrated fluorescence intensity *vs*. time traces (Red) of functional Wnt3A-ATTO680 physisorbed onto a clean glass coverslip (Inset, scale bar 5 μM). Analysis was performed using a modified version of the change-point algorithm (2) (Blue) implemented in MatLab. The vast majority of all molecules displayed one-step photobleaching (210/214) imaged.

Supplementary Figure 2: ATTO680-NHS labelling abolishes Wnt3A activity.

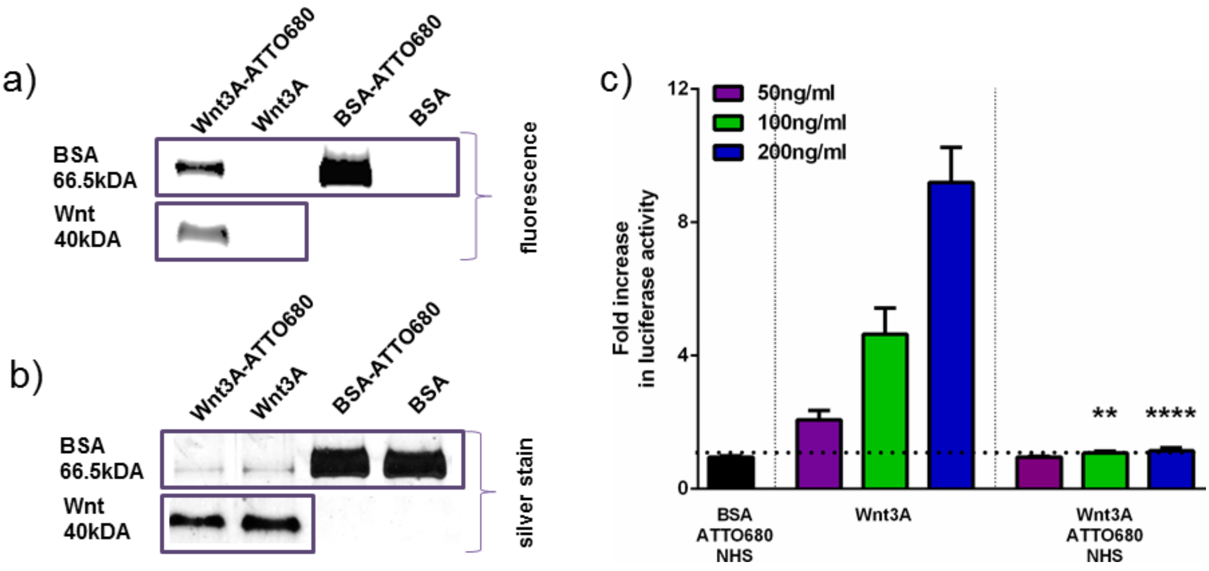

Supplementary Figure 2: Wnt3A protein can be labelled with an NHS-ester derivative of ATTO680 (a) as indicated by imaging on a fluorescent gel reader. b) Representative sliver-stained protein gel. In contrast to ATTO680-maleimide labelling (see Figure 1, main manuscript) ATTO680-NHS labelling abolishes Wnt3A activity in reporter cell assays (c, **p<0.01; ****p<0.0001).

Parameters used for the data analysis using the Single-Dye tracking tool

Analysis was performed using the previously published analysis tool,[1] in this specific case the following parameters were used are listed below:

parameters.time = 30; %(for S2R+ cells, 20 for S2 cells)
parameters.PixelSize =160; %Pixel Size of instrument in nm
parameters.initialthreshold = 5;
parameters.SNR = 3;
parameters.max_spot_size =4; %Maximum Spot Size [radius in pixel]
parameters.minLength = 5; %defines minimal length of tracks to be kept [frames]
parameters.max_step = 3; %defines maximal distance at which 2 spots are linked in subsequent frames [pixels]
parameters.memory = 0; %this is the number of time steps that a particle can be 'lost' and then recovered again [frames]
parameters.step = 5; %Number of points for calculating single MSD and for fit the single trajectories
parameters.n_fit = 5; %Number of points for fitting the ensemble plot
parameters.lobject = 1.5;
parameters.lnoise = 1;
parameters.pkfnd_sz = 0.5;
parameters.cntrd_sz = 2.5;

To test the validity of our analysis, we used Monte-Carlo simulations to generate data of two dimensional Brownian diffusers with known diffusion coefficients, diffraction-limited size, and the same signal-to-background sampled from a distribution levels determined from our experimental data. These simulated data were analysed using the same track detection and fitting protocol of the real data, an example is which is included at Suppl. Movie 6.

Supplementary Figure 3: Localisation Precision of representative data

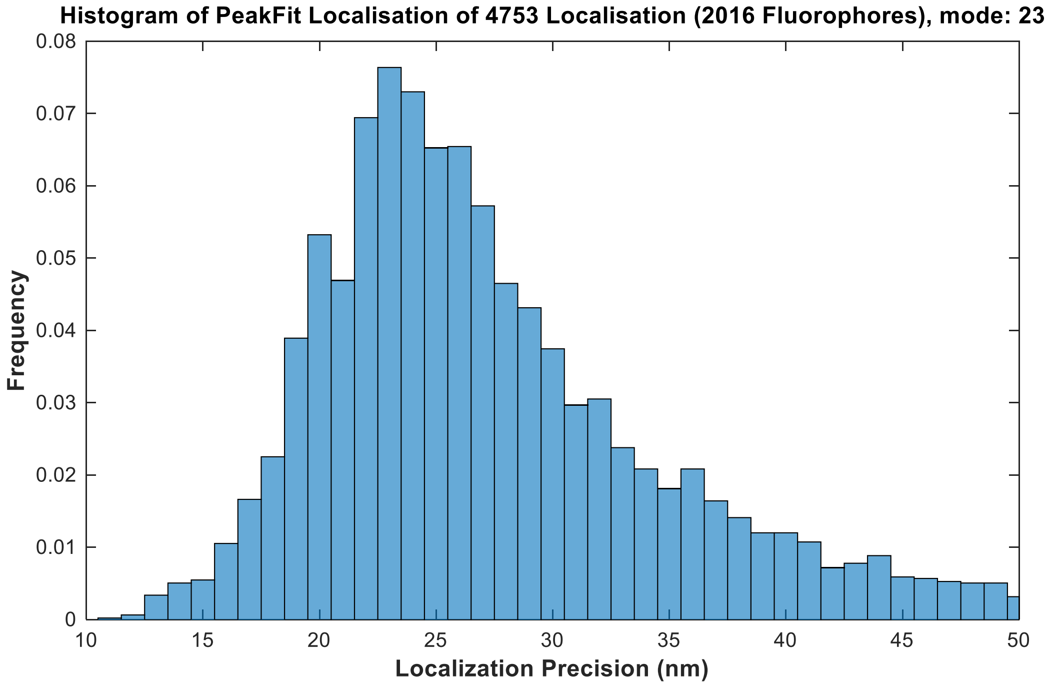

Supplementary Figure 3. Localisation precision was determined using the freely available localization software PeakFit (http://www.sussex.ac.uk/gdsc/intranet/microscopy/imagej/smlm_plugins) from S2 cell diffusion data (4753 Localisation of 2016 Fluorophores).

Simulation of single-particle diffusion

Single-particle diffusion was simulated using the MatLab package ICSMatLab (www.cellmigration.org/resource/imaging/icsmatlab/ICSTutorial.html). This package generates image sequences of Gaussian spots diffusing. Normally distributed signal and background noise is added at to match the quality of the experimental data. The spots are translated by step sizes sampled from a normal distribution based on a specified diffusion coefficient. This code was modified to create Gaussians with subpixel localization. Multiple simulations with different diffusion coefficients were run and added together to generate image sequences representing multiple diffusing populations.

It has been known that MSD is influenced not only by static errors but also by dynamic errors due particle moving while acquisition (3-7). To accommodate for this motion blur we subsampled simulated by a factor of ten. The factor of ten was chosen, so that the MSD during a subsampled frame at the given diffusion coefficient would be less than the localisation precision. The frames were then summed and signal and background values as well as their variances were adjusted to be comparable or slightly poorer than real data (see Suppl. Movie 6).

Simulation (mean +/- StDev): Background: 1230 +/- 250, Signal: 1933 +/- 519

Data (mean +/- StDev): Background: 1128+/- 191, Signal: 1731 +/- 511

The simulated data gave a mean track length of 8, with a mean SNR over the tracks of 5.8 (real data: mean track length: 12, SNR: 9.2).

Supplementary Figure 4: Freely diffusing Brownian motion of simulated particles

The MSD curve was fitted accommodating static and dynamic errors (3-7) in the form of:

$$< x^2 >= 4Dnt_e + 4\,\sigma^2 - 4/3Dt_e$$

With D being the diffusion coefficient, σ the localization precision of motion blurred particles, n the number of frames and t_e the exposure time.

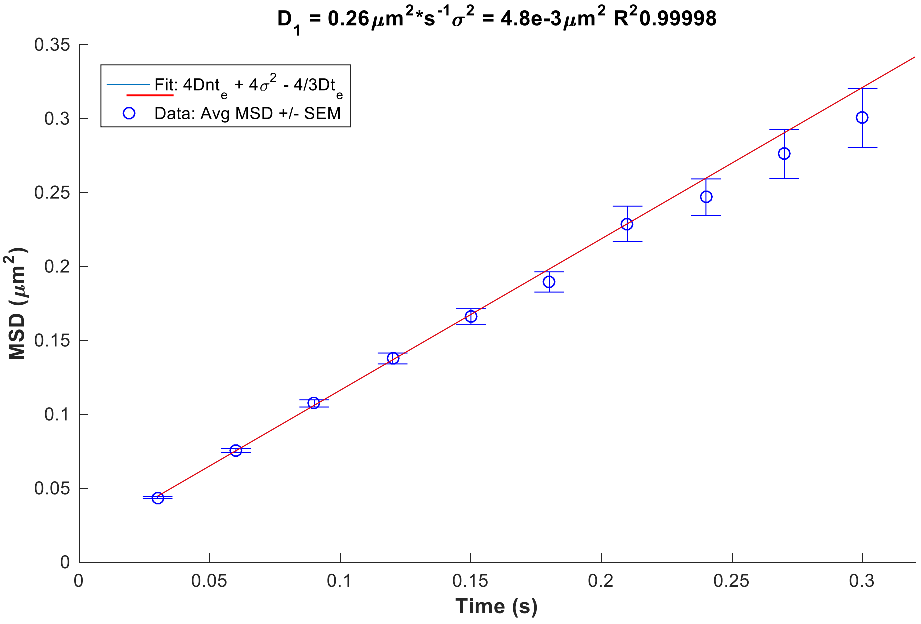

Supplementary Figure 4. MSD Analysis of simulated Data of freely diffusing, motion blurred particles with D = 0.3 $\mu m^2 s^{-1}$, 753 tracks, mean track SNR = 5.8, mean track length 8 at 30 ms exposure and 160 nm pixel size. Plotted is the average MSD curve with SEM as well as the linear fit (red).

Supplementary Figure 5: Wnt3A proteins stay longer associated with receptor expressing S2R+ cells than receptor free S2 cells.

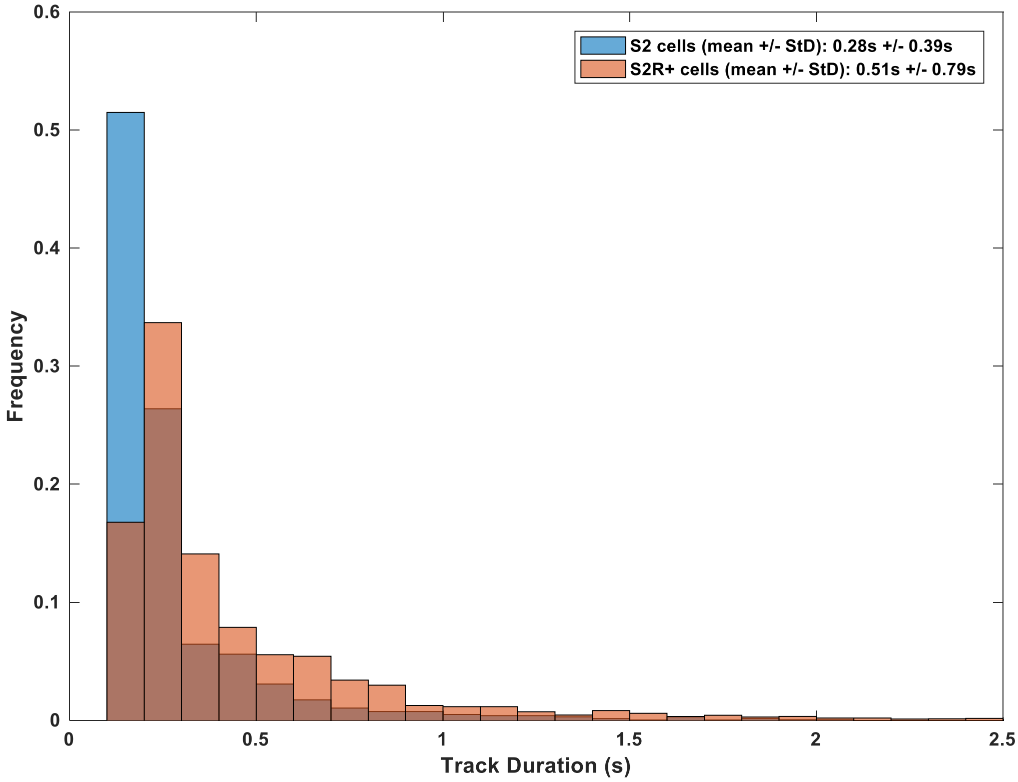

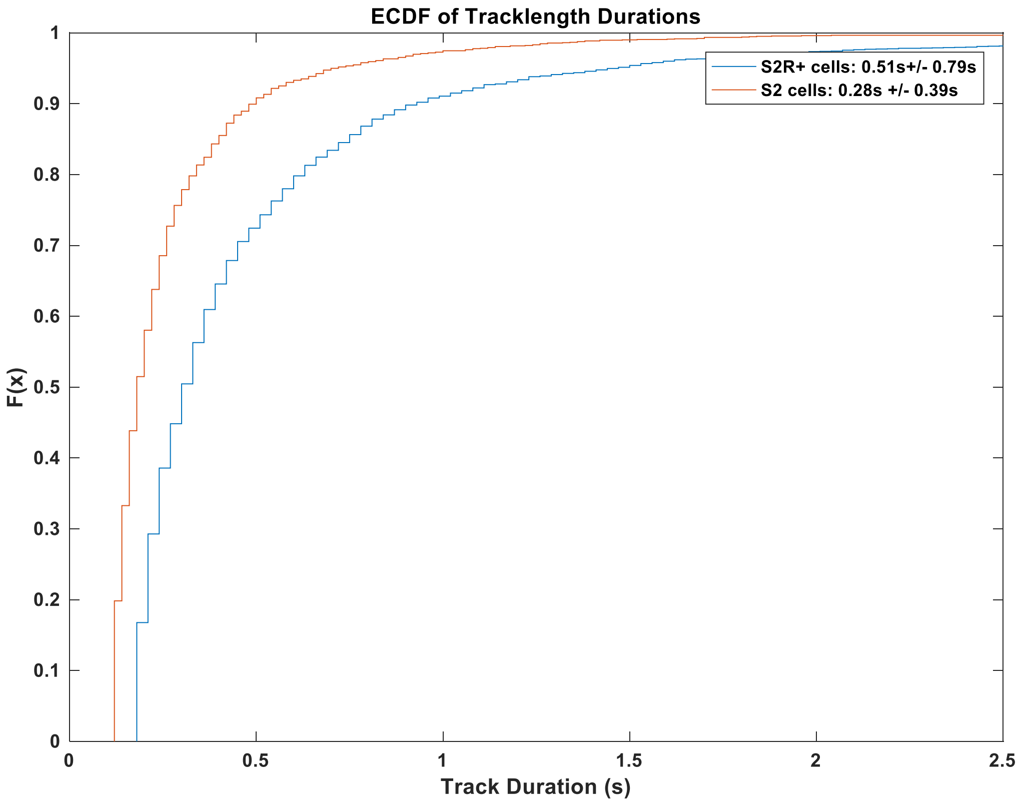

Supplementary Figure 5. Wnt3A Protein stays longer in contact with S2R+ cells than receptor free S2 cells. Shown is the track length distribution of S2 cells (2016 tacks, 5 cells, mean SNR: over track: 9.2) and S2R+ cells (3008 tracks, 8 cells, mean SNR over tracks: 8.7)

Supplementary Figure 6: Continuous replenishment of diffusing Wnt3A proteins on cell membrane.

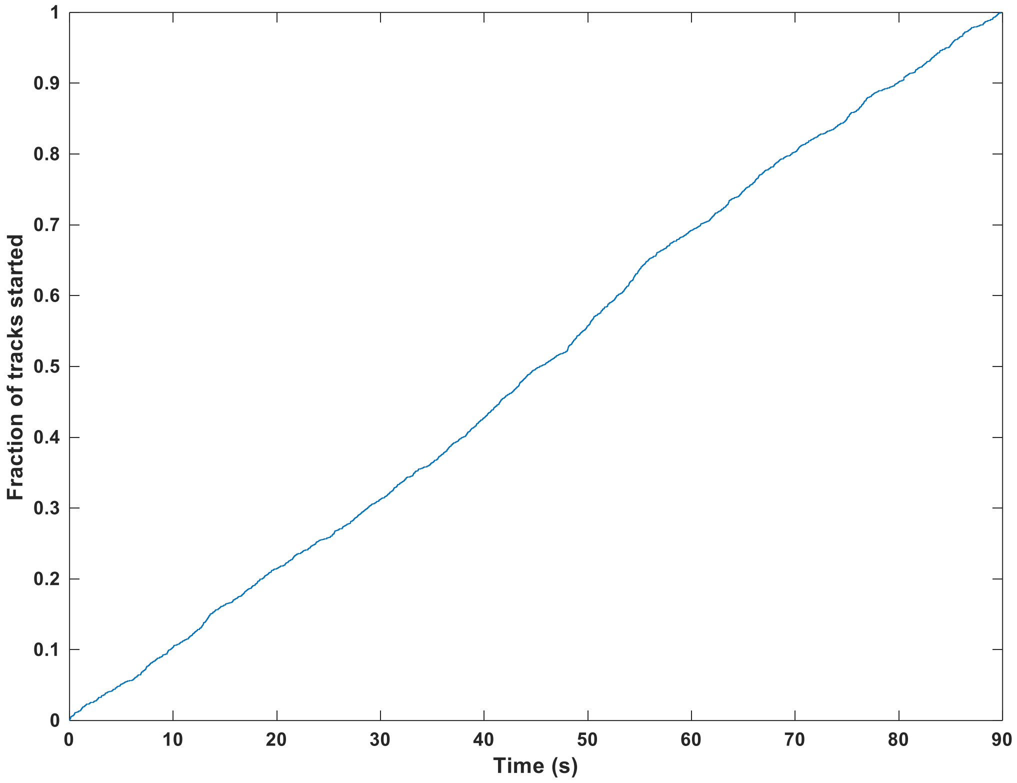

Supplementary Figure 6. Track fraction as a function of time. The rate at which single tracks start is uniform over the acquisition time indicating a constant binding and unbinding of Wnt3A proteins to the membrane. Shown here are the fractions of tracks started in each frame in a S2 cell (2108 tracks).

References

1. PL Weimann, L., Ganzinger, K.A., McColl, J., Irvine, K.L., Davis, S.J., Gay, N.J., Bryant, C.E., Klenerman, D. A Quantitative Comparison of Single-Dye Tracking Analysis Tools Using Monte Carlo Simulations (2013) PLoS ONE, 8 (5), art. no. e64287,

2. Watkins, L.P., Yang, H. Detection of intensity change points in time-resolved single-molecule measurements (2005) Journal of Physical Chemistry B, 109 (1), pp. 617-628.

3. A. J. Berglund, Statistics of camera-based single-particle tracking, Phys. Rev. E 82, 011917 (2010).

4. T. Savin and P. S. Doyle, Static and dynamic errors in particle tracking microrheology, Biophys. J. 88, 623 (2005).

5. X. Michalet and A. J. Berglund, Optimal diffusion coefficient estimation in single-particle tracking, Phys. Rev. E 85, 061916 (2012).

6. X. Michalet, Mean square displacement analysis of singleparticle trajectories with localization error: Brownian motion in an isotropic medium, Phys. Rev. E 82, 041914 (2010).

7. Mikael P. Backlund, Ryan Joyner and W. E. Moerner, Chromosomal locus tracking with proper accounting of static and dynamic errors, Phys. Rev E 91, 062716 (2015).
