# Supplementary material for: Single-Molecule Imaging of Wnt3A Protein Diffusion on Living Cell Membranes
Source: Biophys J. 2017 Dec 19;113(12):2762–7. doi: 10.1016/j.bpj.2017.08.060 (PMC5925569; doi:10.1016/j.bpj.2017.08.060)
Supplement: Document S2. Article plus Supporting Material [file mmc7.pdf]

Biophysical *Journal*
Article

Biophysical Society

Single-Molecule Imaging of Wnt3A Protein Diffusion on Living Cell Membranes

Anna Lippert,[1] Agnieszka A. Janeczek,[2] Alexandre Fürstenberg,[3,4] Aleks Ponjavic,[1] W. E. Moerner,[3] Roel Nusse,[5] Jill A. Helms,[6] Nicholas D. Evans,[2,*] and Steven F. Lee[1,3,*]
[1]Department of Chemistry, University of Cambridge, Cambridge, United Kingdom; [2]Centre for Human Development, Stem Cells and Regeneration, Institute for Life Sciences, University of Southampton, Southampton, United Kingdom; [3]Department of Chemistry, Stanford University, Palo Alto, California; [4]Department of Inorganic and Analytical Chemistry, University of Geneva, Quai Ernest-Ansermet 30, Genève, Switzerland; [5]Department of Developmental Biology, Stanford University, Palo Alto, California; and [6]Division of Plastic and Reconstructive Surgery, Stanford University, Stanford, California

ABSTRACT Wnt proteins are secreted, hydrophobic, lipidated proteins found in all animals that play essential roles in development and disease. Lipid modification is thought to facilitate the interaction of the protein with its receptor, Frizzled, but may also regulate the transport of Wnt protein and its localization at the cell membrane. Here, by employing single-molecule fluorescence techniques, we show that Wnt proteins associate with and diffuse on the plasma membranes of living cells in the absence of any receptor binding. We find that labeled Wnt3A transiently and dynamically associates with the membranes of *Drosophila* Schneider 2 cells, diffuses with Brownian kinetics on flattened membranes and on cellular protrusions, and does not transfer between cells in close contact. In S2 receptor-plus (S2R+) cells, which express Frizzled receptors, membrane diffusion rate is reduced and membrane residency time is increased. These results provide direct evidence of Wnt3A interaction with living cell membranes, and represent, to our knowledge, a new system for investigating the dynamics of Wnt transport.

INTRODUCTION

Wnt proteins are a family of secreted growth factors that are conserved throughout the animal kingdom (1,2). This long evolutionary history is reflected in their involvement in a wide range of mammalian developmental and disease processes. Wnt signaling is necessary for normal development (3), whereas dysregulated Wnt signaling can cause cancer (4).

Although the intracellular trafficking of Wnt proteins and downstream effects of receptor engagement are well studied, much less is known about their extracellular transport. All Wnt proteins (apart from *Drosophila* WntD) have been found to be lipidated (5,6); a modification that is necessary for the intracellular transport, secretion, and activity of Wnt proteins (7). Lipid modifications are unusual in secreted proteins—lipidation might be expected to prevent their movement in the aqueous extracellular space. But in contrast to this, Wnt proteins have been shown to be able to exert effects distant from the site of their production (8). This indicates that there are likely to be controlled mechanisms for Wnt protein transport, evidence for which is emerging from several recent studies. For example, Wnt protein activity is preserved by heparin sulfate proteoglycans (9), which are components of serum and are associated with many cell membranes. Alternatively, Wnt proteins may be transported by association with lipoproteins (10) or other specialist transport proteins in the extracellular space (11–13). Finally, Wnt proteins may be stabilized by direct interactions with biomembranes. Synthetic liposomal carriers can preserve Wnt3A activity in aqueous media and in the absence of other added proteins (14–16), indicating membrane binding. In vivo, Wnt proteins have been shown to be carried by exosomes in some circumstances (17), and they may activate signaling through direct cell-cell contact, either via delivery on long cellular processes called cytonemes (18,19) or by intimate membrane contact in stem cell niches (20). In this latter study, Wnt3 protein was shown to be directly transferred from the surface of secreting cells to receiving cells, a process that is dependent on Frizzled (Fz) expression on the latter cells. Although interaction with Evi/Wls is required for presentation of Wnt protein at the cell membrane of secreting cells (21), whether Wnt proteins can bind to cell membranes in the absence of Fz

Submitted April 6, 2017, and accepted for publication August 2, 2017.
*Correspondence: n.d.evans@soton.ac.uk or sl591@cam.ac.uk
Anna Lippert and Agnieszka A. Janeczek contributed equally to this work.
Editor: Joseph Falke.
https://doi.org/10.1016/j.bpj.2017.08.060
© 2017 The Authors.
This is an open access article under the CC BY license (http://creativecommons.org/licenses/by/4.0/).

or Evi/Wls, or require Fz for delivery from carriers remains unproven, primarily due to the lack of suitable methods to investigate this at the single-receptor level.

MATERIALS AND METHODS

Wnt labeling

Wnt3A protein was either purchased from R&D Systems (5036-WN/CF) or purified from the supernatant of S2 cells stably expressing the murine Wnt3A protein by blue sepharose and immobilized metal affinity chromatography, followed by gel filtration and heparin cation exchange according to the method of Willert (22). For labeling, ATTO680-maleimide (04971-1MG; Sigma-Aldrich, St. Louis, MO) or ATTO680-NHS (75999-1MG; Sigma-Aldrich) was diluted to a final concentration of 1.5 μM and added to purified Wnt protein with a concentration of 20 μg/mL in phosphate buffered saline (PBS) containing 1% (w/v) 3-[(3-cholamidopropyl)dimethylammonio]-1-propanesulfonate, resulting in a stoichiometric molar ratio of 3:1. The reaction was allowed to proceed for 1 h at room temperature. After this, the mixture was passed three times through a micro bio-spin six-column equilibrated with PBS containing 1% 3-[(3-cholamidopropyl)dimethylammonio]-1-propanesulfonate to exclude unbound dye molecules using a centrifuge (Fisher Minispin, CFA-165-010L at 13,500 Rpm). To test for successful labeling, between 50 and 500 ng of dye-labeled or unlabeled Wnt3A (R&D Systems carrier-free protein) were resolved on a 10% polyacrylamide ProtoGel gel (National Diagnostics) using Bio-Rad system. The fluorescent tag on the protein within the gel was imaged on the LI-COR Odyssey system. Sizing was achieved using a fluorescent sizing ladder (Precision Plus Protein All Blue Standards; Bio-Rad, Hercules, CA). The quantity of loaded protein was assessed by subsequent staining of the gel with a Proteo Silver Stain Kit (Sigma). Confirmation of the 40 kDa band as Wnt was conducted via Western blot after transferring the protein onto a polyvinylidene fluoride membrane (Merck Millipore, Burlington, MA) and detection performed using a Wnt3A antibody (Ab172612; Abcam), IgG HRP (Ab6721; Abcam), and Immobilon Western Chemiluminescent HRP Substrate (Merck Millipore) on a Versadoc reader (Bio-Rad).

Activity assays

The luciferase assay was performed on a 3T3 mouse embryonic fibroblast cell line (Enzo Life Sciences), modified to expresses the firefly luciferase reporter gene under the control of Wnt-responsive promoters (TCF/LEF). Briefly, cells were seeded onto white, clear-bottomed, 96-well plates, at 1.5×10^4 cells/well in 50 μL assay medium (Enzo Life Sciences) and incubated with Wnt3A preparations at 50, 100, and 200 ng/mL for ~18 h. Next, 100 μL per well of Steady-Glo luciferase reagent (Promega) was added for ~10 min and the chemiluminescence signal was read (0.1 s per well) on a Varioscan Flash microplate reader (Thermo Scientific, Waltham, MA). To control for cell count, cell lysates were analyzed for double-stranded DNA content using PicoGreen reagent (Thermo Fisher), according to manufacturer's protocol.

Cell culture

Drosophila S2 cells were cultured in suspensions in capped flasks at room temperature in Schneider's *Drosophila* Medium (Invitrogen, Carlsbad, CA) supplemented with 10% heat inactivated, insect cell tested, fetal bovine serum (Sigma) and penicillin/streptomycin. Cells were passaged at a dilution of 1:20 every week, with the addition of fresh medium. S2 receptor-plus (S2R+) cells were grown in the same medium, but as adherent cells were passaged every 4–5 days at a dilution of 1:5. Cells were detached from tissue culture substrata before passaging by gentle mechanical agitation.

For microscopy experiments, chamber slides were coated with 0.5 mg/mL filter sterilized concanavalin A for 1 h before S2 and S2R+ cells were plated at a density of 1000/cm^2. Medium was replaced after 1 h to remove nonadherent cells.

For live cell imaging, cells were thoroughly washed with PBS before incubation in a modified Krebs Ringer's phosphate buffer made in-house (136 mM NaCl, 4.7 mM KCl, 1.25 mM $MgSO_2$, 1.25 mM $CaCl_2$, 5 mM NaH_2PO_4, 2 mM $NaHCO_2$, 10 mM glucose, and 25 mM HEPES (pH 7.2)) supplemented with 1% serum.

Chamber slides were positioned on the stage of the microscope and suitable cell areas were selected by white-light microscopy. At given time points, labeled or unlabeled proteins (Wnt3A and bovine serum albumin (BSA)) or dye alone were added at final concentrations of 1–2 nM (0.04–0.08 μg/mL). In some cases, proteins were denatured before addition by heating at 90°C for 10 min.

Tracking experiments

White-light transmission and single-molecule fluorescence images were acquired with a modified fluorescent microscope based on an Olympus IX71 inverted microscope, equipped with an infinity-corrected oil immersion objective (Olympus UPlanApo, ×100, 1.4 NA), operating in highly inclined and laminated optical sheet imaging mode (23) to reduce the excitation volume, and detected on a 512 × 512 pixel EMCCD (Andor I-Xon2, 897) at a rate of 20–30 ms per frame for Wnt3A imaging. The general epifluorescence setup has been described previously (24); here, the filters used were a dichroic mirror (Di01-R635-25x36; Semrock) and a 635 nm long pass filter (BLP01-635R-25; Semrock). Laser excitation was provided by a 635 nm solid-state laser (Blue Sky Research, FTEC2-635-V50PFM, 638 nm FiberTECII laser) at a power density of ~250 W/cm^2.

Tracking analysis

Tracking analysis of single Wnt proteins was accomplished using previously published single-molecule tracking software (25). This custom-written MATLAB code uses an interactive data language particle-tracking function defined previously (26). The tracking analysis determines the diffusion coefficient from both the mean-square displacement and via jump distance analysis. For a detailed description of the specific details, please refer to the Supporting Material.

To test the validity of our analysis, we used simulated data. Here we used the MATLAB package ICSMatlab (http://www.cellmigration.org/resource/imaging/icsmatlab/ICSTutorial.html) to generate movies of two-dimensional Brownian diffusers with specified diffusion coefficients, diffraction-limited size, and the same signal-to-noise distribution levels as our experimental data. These simulated data (see Movie S1) were analyzed using the same track detection and fitting protocol as the real data.

RESULTS AND DISCUSSION

We employed protein labeling and single-molecule fluorescence microscopy to image the interaction of Wnt3A protein with living cell membranes at high temporal resolution. There are few reports of active Wnt proteins produced by fusion of fluorescent proteins (18,27), possibly because they often do not retain activity on subsequent expression and posttranslational processing. However, Wnt3A retained activity after coupling to carboxylic acid-modified microbeads (28), suggesting covalent dye attachment as a possible method for Wnt protein labeling. We reacted carrier-free Wnt3A protein with maleimide or

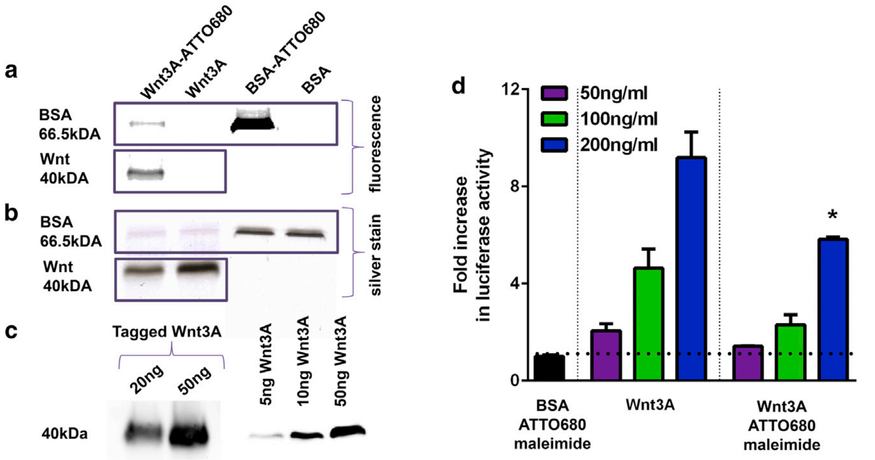

FIGURE 1 (*a*) ATTO680-maleimide labeling of Wnt3A protein causes moderate decreases in activity. Wnt3A protein was tagged and run on SDS-PAGE gels and imaged under a fluorescence illumination reader. Fluorescent bands were visible at masses of 66.5 kDa, corresponding to a BSA-only control, and at 40 kDa, the known mass of Wnt3A. (*b*) Presence of protein was confirmed using silver staining. (*c*) Western blotting confirmed that the labeled band at 40 kDa was Wnt. (*d*) ATTO680-maleimide tagging caused decreases in activity in luciferase reporter assays (*$p < 0.05$).

N-hydroxysuccinimide (NHS) ester functionalized fluorophores. Wnt3A protein was successfully labeled by ATTO680-maleimide, as confirmed by polyacrylamide gel electrophoresis and Western blotting (Fig. 1, *a–c*). Labeled protein was evident as a fluorescent band at a mass size of ~40 kDa. Another predominant fluorescent band was evident at a molecular mass of ~66 kDa, which is likely due to BSA, as suggested by the presence of a band of equal mass in control labeling experiments. Single-molecule bleaching analysis revealed >98% (210 out of 214 molecules) of labeled proteins bleach within a single imaging step, suggesting most of protein was tagged only once with the fluorophore (Fig. S1). We then tested the activity of the labeled protein preparations by incubating either labeled proteins or the control protein (subject to the same labeling conditions) with a reporter cell line that produces luciferase under the control of a Wnt-responsive promoter. Protein labeling with ATTO680-maleimide reduced Wnt3A activity by 37 ± 9% ($p < 0.05$, Fig. 1 *d*). Alexa Fluor 647-maleimide and ATTO680-NHS also successfully labeled Wnt3A protein, but whereas the former had no significant effect on protein activity, the latter entirely abolished its activity (Fig. S2). This suggests that labeling of protein at free amine groups is detrimental to Wnt3A activity, whereas cysteine labeling is less so. There is evidence to indicate that all cysteines in Wnt proteins are involved in disulfide bridges (25). Nevertheless, we were able to label Wnt3A in nonreducing conditions with significant retention of protein activity. This suggests that there must be free cysteine residues present in Wnt3A preparations. It is likely that the cysteines labeled are those that have a low impact on Wnt3A activity when mutated, for example, those cysteines involved in the formation of hairpin 1. Future studies may address the location of Wnt3A labeling by, for instance, liquid chromatography tandem mass spectrometry (6).

Having established a method of labeling Wnt3A protein, we next tested whether it can associate with cell membranes in the absence of Wnt receptors. Schneider 2 (S2) cells are derived from *Drosophila* embryos, exhibit macrophage-like behavior, and do not express Fz or Wnt proteins (29). As S2 cells are poorly adherent, glass coverslips were precoated with concanavalin A, a lectin which causes S2 cells to adhere and flatten, with a rounded morphology in the vicinity of the nucleus and a large, round, flattened cytoplasmic "skirt" (Fig. 2 *a*) (30). These latter areas provided an excellent two-dimensional platform for measuring the diffusion of molecules in real-time using highly inclined and laminated optical sheet (pseudo-total internal reflection fluorescence) microscopy (23).

After addition of labeled Wnt3A protein, at a physiological concentration of 1–2 nM (0.04–0.08 μg/mL), single diffusing molecules were evident on the plasma membrane (Fig. 2, *a–c*; Movie S2 (left)). Using previously published, single-molecule tracking software (31), the kinetics were analyzed and found to be consistent with a Brownian diffuser model (Fig. S4), which accommodates for static and dynamic errors that are present in camera-based motion tracking (32). The fitting function illustrates the unhindered random movement of the labeled protein on the cell membrane yielding a diffusion coefficient (*D* value ± fit error) of 0.33 ± 0.01 $\mu m^2 s^{-1}$ from a linear mean-square displacement plot with an average track length (mean track length ± SD of 0.27 ± 0.39 s for S2) (Fig. S5).

To test whether the presence of Wnt receptors affected diffusion kinetics of membrane-associated Wnt3A protein, we compared S2 diffusion (Fig. 2 *d*) with diffusion on SR2$^+$ cells (Fig. 2 *e*), a similar cell line that expresses both DFz1 and DFz2 (33). Despite species differences, there is significant promiscuity in Wnt and Fz interaction, and mammalian Wnt3A has been shown to bind to *Drosophila* Fz proteins (34). In S2R+ cells, we observed a significant decrease in the diffusion coefficient (*D* value ± fit error) of 0.18 ± 0.01 $\mu m^2 s^{-1}$ (Fig. 2 *f*) and an increase in track length (mean track length ± SD) of 0.51 ± 0.79 s (Fig. S5). In contrast, with the addition of either ATTO680 alone or ATTO680-labeled BSA, we observed

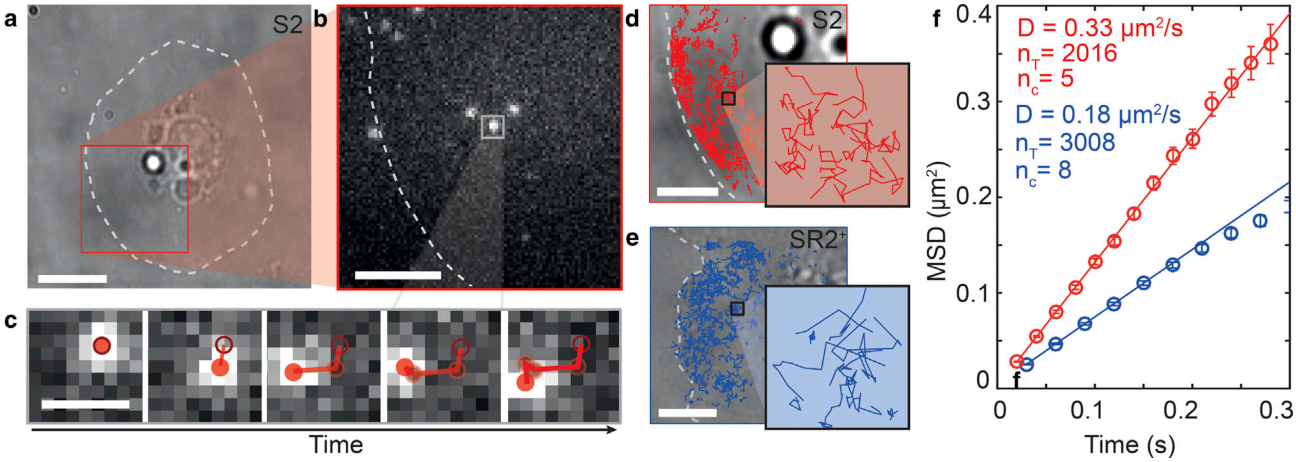

FIGURE 2 Wnt diffusion is slowed by the presence of the Wnt receptor in S2R+ cells. Fluorophore-labeled Wnt protein was imaged on S2 (*red*) and S2R+ (*blue*) cells using highly inclined and laminated optical sheet microscopy ((*a*) shows a white-light image and (*b*) shows a single-molecule fluorescence image). (*c*) A tracking algorithm (26) was used to link fluorescent puncta (*red circles*, starting (*dark red*) and current position (*filled*)) in consecutive frames as described in Materials and Methods (scale bars, 1 μm), resulting in tracks with a localization precision of ~23 nm (Fig. S3). (*d* and *e*) Overlay of tracks with white-light images (scale bars, 5 μm). (*f*) The ensemble diffusion coefficient was determined by fitting a linear function, which considers static and dynamic errors (32; Fig. S4), to the mean-square displacement (MSD) values versus time. Shown are average MSD values for S2 (*red*) and S2R+ (*blue*) cells with mean $\pm$ SE, as well as the linear corresponding fit.

no isolated diffusers on the membrane of these cells (Movie S3). Heat inactivation (10 min at 90°C) of labeled Wnt3A protein completely abolished the appearance of diffusers on membranes, suggesting that active Wnt3A protein is responsible for the diffusion observed (Movie S4). These results are consistent with a decrease in the diffusion speed of Wnt3A once bound to a receptor, and consistent with other membrane proteins (35). However, we cannot rule out that this may be attributed to other differences in the cell lines, including cytoskeletal differences, membrane lipid composition, or membrane protein density (36,37). Taken together, we conclude that Wnt3A associates with and diffuses unhindered on the surface of living cell membranes, the presence of a receptor both increases the residency time of this interaction and reduces the diffusion coefficient by ~two-fold in both cases.

In addition to the quantitative kinetic information, the single-molecule tracking analysis has allowed extraction of several behaviors of Wnt molecules that could not be determined using ensemble-averaged techniques, discussed below.

Wnt3A dynamically binds onto and off the cell membrane

Analyzing the starting position of tracks during the course of a single-molecule tracking experiment revealed a spatially independent probability for the tracks starting within the laser excitation zone (Fig. 3 *a*). Typically in single-molecule tracking experiments, new labeled molecules can only diffuse in from the periphery of the excitation zone, tested by periods of inactive laser excitation, which allows new molecules to diffuse into the zone before photobleaching occurs (analogous to fluorescence recovery after photobleaching experiments); this was not observed. Instead, single Wnt molecules consistently began their tracks at any point within the excitation zone (Movie S2 (right)). These data suggest a mechanism where exogenous Wnt3A is in equilibrium between cell membrane binding and either free diffusion in solution or binding to soluble factors. To prevent Wnt precipitation and to preserve its activity, serum was added at 1% to all experiments. Serum is known to contain stabilizing proteins that bind Wnt, such as afamin (12), heparan sulphate proteoglycan (9), or lipoproteins (10). In addition to this, a linear relationship of the single-molecule track starting frame versus time (Fig. S6) was observed in the 2108 tracks examined, which suggests a continual replenishment of single diffusers (from solution) over the course of the experiment, analogous to the commonly used Point Accumulation for Imaging in Nanoscale Topography technique (38).

Wnt3A does not transfer between adjacent S2 cells

Another possible mechanism for extracellular Wnt transport is direct membrane diffusion from the surface of one cell to another (20,39,40). To test this, we grew dense cultures of S2 cells to a level where the "skirts" began to come into contact/close apposition (see Fig. 3 *b*). Labeled Wnt3A was allowed to diffuse on the surface and we observed diffusion at the border between cells. Despite extensive efforts, we did not observe labeled Wnt3A diffusing across the membrane boundary from cell to cell (Movie S5). Fig. 3 *b* demonstrates a representative experiment of single-molecule tracks at a cell-cell boundary overlaid onto the white-

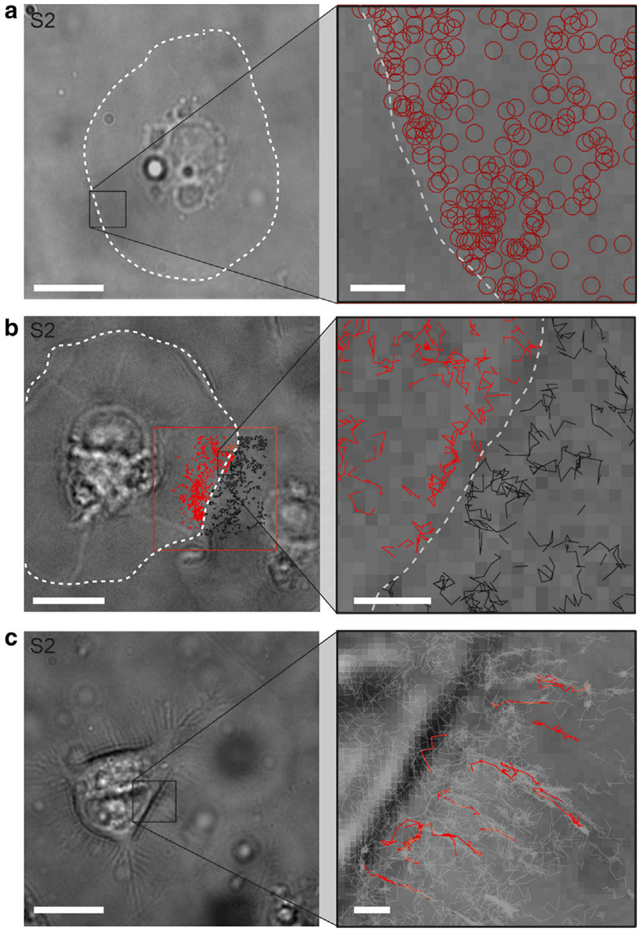

FIGURE 3 Properties of Wnt S2 membrane associations. (*a*) Wnt3A binds from solution. The starting positions of Wnt tracks (*dark red circles*) are evenly distributed on the plasma membrane (*white dashed line*) of S2 cells demonstrating the probability of single-molecule trajectories occurring is spatially independent. (*b*) Wnt3A does not transfer between cells. Wnt protein tracks were not found to be crossing cell boundaries of adjacent cells (*white dashed line*) on S2 cells. The red box indicates the analyzed area. (*c*) Wnt3A diffuses along cellular processes. Single Wnt3A molecules were also found along cellular processes, suggesting free diffusion along these structures. Wnt tracks are shown in gray with some highlighted tracks in red. Scale bars: left, 10 μm; right, 1 μm.

light image of the cell. No trajectory was observed in which the protein diffused from one cell to another over a time course of ~15 min. Although S2 cells are a suitable model for generalized measurements of membrane diffusion of labeled proteins, further experiments would be necessary to confirm whether Wnt proteins may be transmitted between cells in other situations, such as on intracellular membranous bridges between adjacent cells (41) or via hemifused lipid bilayers at tight junctions (39,42).

Wnt3A diffuses along cellular processes

Accumulating evidence suggests that Wnt ligands can be mobilized and transferred on the tips of filopodia (18,19). Although spread S2 cells do not readily form filopodia, in retracting cells, membrane protrusions are visible (Fig. 3 *c*). In such protrusions we often saw diffusion of Wnt3A protein, indicating that membrane-bound Wnt3A is capable of free transport on filopodia. Although it is likely that specific mechanisms control the presentation of secreted Wnt ligand at the tips of these protrusions, based on these data, it is also possible that membrane-localized Wnt ligands freely diffuse along filopodia to activate signaling in cells that they contact. This may be regulated by membrane composition, as has been recently shown for Wnt3 (43).

Single-molecule fluorescence microscopy is a powerful tool to study Wnt protein dynamics, with which we have quantitatively demonstrated unhindered diffusion of active protein on the surface of living cells. We find that Wnt3A diffused two-fold more slowly and for twice as long in the presence of cells expressing its receptor. Further, we have determined that the transport mechanism is likely based on a binding-unbinding equilibrium of the protein with the plasma membrane and the surrounding solution. It is expected further that single-molecule studies will reveal a more complex understanding of both Wnt proteins and cell signaling in general.

SUPPORTING MATERIAL

Supporting Materials and Methods, six figures, and five movies are available at http://www.biophysj.org/biophysj/supplemental/S0006-3495(17)31128-1.

AUTHOR CONTRIBUTIONS

S.F.L. and N.D.E. designed the experimental plan. A.A.J., A.L., A.F., S.F.L., and N.D.E. performed experiments and analyzed the data. A.P. provided the tracking simulation code. A.L., A.A.J., A.F., A.P., W.E.M., R.N., J.A.H., N.D.E., and S.F.L. wrote the article and/or contributed to revisions.

ACKNOWLEDGMENTS

We thank the Royal Society for the University Research Fellowship of S.F.L. (UF120277). We thank the Medical Research Council for funding A.A.J's studentship (MR/J004103/1). We also thank the Bone Research Society, UK for a Barbara Mawer travel award and the Tissue and Cell Engineering Society for a Short Scientific Mission award to A.A.J. This work was supported in part by the National Institute of General Medical Sciences grant R01GM086196 (to W.E.M).

REFERENCES

1. Clevers, H., and R. Nusse. 2012. Wnt/β-catenin signaling and disease. *Cell*. 149:1192–1205.
2. Holstein, T. W. 2012. The evolution of the Wnt pathway. *Cold Spring Harb. Perspect. Biol.* 4:a007922.
3. van Amerongen, R., and A. Berns. 2006. Knockout mouse models to study Wnt signal transduction. *Trends Genet.* 22:678–689.
4. Bienz, M., and H. Clevers. 2000. Linking colorectal cancer to Wnt signaling. *Cell*. 103:311–320.

5. Takada, R., Y. Satomi, …, S. Takada. 2006. Monounsaturated fatty acid modification of Wnt protein: its role in Wnt secretion. *Dev. Cell.* 11:791–801.
6. Willert, K., J. D. Brown, …, R. Nusse. 2003. Wnt proteins are lipid-modified and can act as stem cell growth factors. *Nature.* 423:448–452.
7. Gross, J. C., and M. Boutros. 2013. Secretion and extracellular space travel of Wnt proteins. *Curr. Opin. Genet. Dev.* 23:385–390.
8. Zecca, M., K. Basler, and G. Struhl. 1996. Direct and long-range action of a wingless morphogen gradient. *Cell.* 87:833–844.
9. Fuerer, C., S. J. Habib, and R. Nusse. 2010. A study on the interactions between heparan sulfate proteoglycans and Wnt proteins. *Dev. Dyn.* 239:184–190.
10. Neumann, S., D. Y. Coudreuse, …, H. Sprong. 2009. Mammalian Wnt3a is released on lipoprotein particles. *Traffic.* 10:334–343.
11. Mulligan, K. A., C. Fuerer, …, R. Nusse. 2012. Secreted Wingless-interacting molecule (Swim) promotes long-range signaling by maintaining Wingless solubility. *Proc. Natl. Acad. Sci. USA.* 109:370–377.
12. Mihara, E., H. Hirai, …, J. Takagi. 2016. Active and water-soluble form of lipidated Wnt protein is maintained by a serum glycoprotein afamin/α-albumin. *eLife.* 5:e11621.
13. Mii, Y., and M. Taira. 2009. Secreted Frizzled-related proteins enhance the diffusion of Wnt ligands and expand their signalling range. *Development.* 136:4083–4088.
14. Morrell, N. T., P. Leucht, …, R. Nusse. 2008. Liposomal packaging generates Wnt protein with In Vivo biological activity. *PLoS One.* 3:e2930.
15. Dhamdhere, G. R., M. Y. Fang, …, J. A. Helms. 2014. Drugging a stem cell compartment using Wnt3a protein as a therapeutic. *PLoS One.* 9:e83650.
16. Janeczek, A. A., E. Scarpa, …, N. D. Evans. 2017. PEGylated liposomes associate with Wnt3A protein and expand putative stem cells in human bone marrow populations. *Nanomedicine (Lond).* 12:845–863.
17. Gross, J. C., V. Chaudhary, …, M. Boutros. 2012. Active Wnt proteins are secreted on exosomes. *Nat. Cell Biol.* 14:1036–1045.
18. Luz, M., S. Spannl-Müller, …, M. Brand. 2014. Dynamic association with donor cell filopodia and lipid-modification are essential features of Wnt8a during patterning of the zebrafish neuroectoderm. *PLoS One.* 9:e84922.
19. Stanganello, E., A. I. H. Hagemann, …, S. Scholpp. 2015. Filopodia-based Wnt transport during vertebrate tissue patterning. *Nat. Commun.* 6:5846.
20. Farin, H. F., I. Jordens, …, H. Clevers. 2016. Visualization of a short-range Wnt gradient in the intestinal stem-cell niche. *Nature.* 530:340–343.
21. Bänziger, C., D. Soldini, …, K. Basler. 2006. Wntless, a conserved membrane protein dedicated to the secretion of Wnt proteins from signaling cells. *Cell.* 125:509–522.
22. Willert, K. H. 2008. Isolation and application of bioactive Wnt proteins. *Methods Mol Biol.* 468:17–29.
23. Tokunaga, M., N. Imamoto, and K. Sakata-Sogawa. 2008. Highly inclined thin illumination enables clear single-molecule imaging in cells. *Nat. Methods.* 5:159–161.
24. Broadhead, M. J., M. H. Horrocks, …, S. G. N. Grant. 2016. PSD95 nanoclusters are postsynaptic building blocks in hippocampus circuits. *Sci. Rep.* 6:24626.
25. MacDonald, B. T., A. Hien, …, X. He. 2014. Disulfide bond requirements for active Wnt ligands. *J. Biol. Chem.* 289:18122–18136.
26. Crocker, J. C., and D. G. Grier. 1996. Methods of digital video microscopy for colloidal studies. *J. Colloid Interface Sci.* 179:298–310.
27. Holzer, T., K. Liffers, …, D. Gradl. 2012. Live imaging of active fluorophore labelled Wnt proteins. *FEBS Lett.* 586:1638–1644.
28. Habib, S. J., B. C. Chen, …, R. Nusse. 2013. A localized Wnt signal orients asymmetric stem cell division in vitro. *Science.* 339:1445–1448.
29. Bhanot, P., M. Brink, …, R. Nusse. 1996. A new member of the frizzled family from Drosophila functions as a Wingless receptor. *Nature.* 382:225–230.
30. Rogers, S. L., U. Wiedemann, …, R. D. Vale. 2003. Molecular requirements for actin-based lamella formation in Drosophila S2 cells. *J. Cell Biol.* 162:1079–1088.
31. Weimann, L., K. A. Ganzinger, …, D. Klenerman. 2013. A quantitative comparison of single-dye tracking analysis tools using Monte Carlo simulations. *PLoS One.* 8:e64287.
32. Backlund, M. P., R. Joyner, and W. E. Moerner. 2015. Chromosomal locus tracking with proper accounting of static and dynamic errors. *Phys. Rev. E Stat. Nonlin. Soft Matter Phys.* 91:062716.
33. Yanagawa, S., J. S. Lee, and A. Ishimoto. 1998. Identification and characterization of a novel line of Drosophila Schneider S2 cells that respond to wingless signaling. *J. Biol. Chem.* 273:32353–32359.
34. Takada, R., H. Hijikata, …, S. Takada. 2005. Analysis of combinatorial effects of Wnts and Frizzleds on beta-catenin/armadillo stabilization and Dishevelled phosphorylation. *Genes Cells.* 10:919–928.
35. Hern, J. A., A. H. Baig, …, N. J. M. Birdsall. 2010. Formation and dissociation of M1 muscarinic receptor dimers seen by total internal reflection fluorescence imaging of single molecules. *Proc. Natl. Acad. Sci. USA.* 107:2693–2698.
36. Frick, M., K. Schmidt, and B. J. Nichols. 2007. Modulation of lateral diffusion in the plasma membrane by protein density. *Curr. Biol.* 17:462–467.
37. Kusumi, A., C. Nakada, …, T. Fujiwara. 2005. Paradigm shift of the plasma membrane concept from the two-dimensional continuum fluid to the partitioned fluid: high-speed single-molecule tracking of membrane molecules. *Annu. Rev. Biophys. Biomol. Struct.* 34:351–378.
38. Lew, M. D., S. F. Lee, …, W. E. Moerner. 2011. Three-dimensional superresolution colocalization of intracellular protein superstructures and the cell surface in live Caulobacter crescentus. *Proc. Natl. Acad. Sci. USA.* 108:E1102–E1110.
39. Laffafian, I., and M. B. Hallett. 2007. Lipid-protein cargo transfer: a mode of direct cell-to-cell communication for lipids and their associated proteins. *J. Cell. Physiol.* 210:336–342.
40. Trimble, W. S., and S. Grinstein. 2015. Barriers to the free diffusion of proteins and lipids in the plasma membrane. *J. Cell Biol.* 208:259–271.
41. Caneparo, L., P. Pantazis, …, S. E. Fraser. 2011. Intercellular bridges in vertebrate gastrulation. *PLoS One.* 6:e20230.
42. Grebenkämper, K., and H.-J. Galla. 1994. Translational diffusion measurements of a fluorescent phospholipid between MDCK-I cells support the lipid model of the tight junctions. *Chem. Phys. Lipids.* 71:133–143.
43. Ng, X. W., C. Teh, …, T. Wohland. 2016. The secreted signaling protein Wnt3 is associated with membrane domains In Vivo: a SPIM-FCS study. *Biophys. J.* 111:418–429.

Biophysical Journal, Volume *113*

Supplemental Information

Single-Molecule Imaging of Wnt3A Protein Diffusion on Living Cell Membranes

Anna Lippert, Agnieszka A. Janeczek, Alexandre Fürstenberg, Aleks Ponjavic, W.E. Moerner, Roel Nusse, Jill A. Helms, Nicholas D. Evans, and Steven F. Lee

Supplementary Material

Single-molecule imaging of Wnt3A protein diffusion on living cell membranes

Supplementary Figure 1. Photobleaching analysis.

Supplementary Figure 2: ATTO680-NHS labelling abolishes Wnt3A activity.

Simulation of single-particle diffusion

Parameters used for the data analysis using the Single-Dye tracking tool

Supplementary Figure 3: Localisation Precision of Data

Supplementary Figure 4: Freely diffusing Brownian motion of simulated Particles

Supplementary Figure 5: Wnt3A proteins stay longer associated with receptor expressing S2R+ cells than receptor free S2 cells.

Supplementary Figure 6: Continuous replenishment of diffusing Wnt3A proteins on cell membrane.

Supplementary Movie 1: Labelled Wnt3A Diffusing on S2 Cells with marked starting positions of the tracks.

Supplementary Movie 2: ATTO680-labelled BSA control on S2 cells.

Supplementary Movie 3: Heat inactivated Wnt3A does not diffuse on S2 cells.

Supplementary Movie 4: Labelled Wnt3A does not transfer across cell boundaries of adjacent S2 cells

Supplementary Movie 5: Simulated *vs.* Real Data

Supplementary Figure 1: Photobleaching Analysis

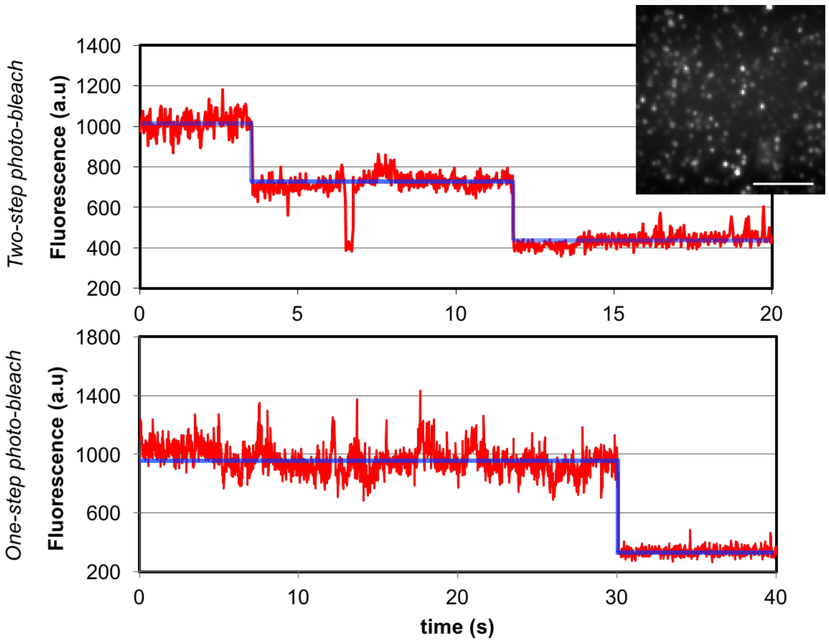

Supplementary Figure 1. Photobleaching analysis. Representative examples of integrated fluorescence intensity *vs*. time traces (Red) of functional Wnt3A-ATTO680 physisorbed onto a clean glass coverslip (Inset, scale bar 5 µM). Analysis was performed using a modified version of the change-point algorithm (2) (Blue) implemented in MatLab. The vast majority of all molecules displayed one-step photobleaching (210/214) imaged.

Supplementary Figure 2: ATTO680-NHS labelling abolishes Wnt3A activity.

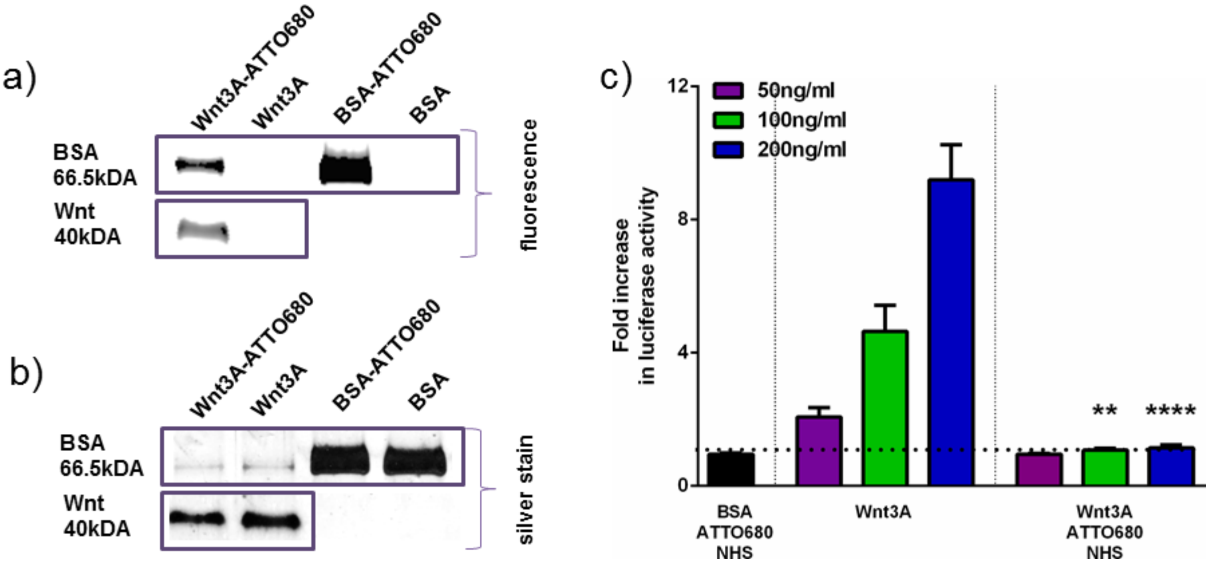

Supplementary Figure 2: Wnt3A protein can be labelled with an NHS-ester derivative of ATTO680 (a) as indicated by imaging on a fluorescent gel reader. b) Representative sliver-stained protein gel. In contrast to ATTO680-maleimide labelling (see Figure 1, main manuscript) ATTO680-NHS labelling abolishes Wnt3A activity in reporter cell assays (c, **p<0.01; ****p<0.0001).

Parameters used for the data analysis using the Single-Dye tracking tool

Analysis was performed using the previously published analysis tool,[1] in this specific case the following parameters were used are listed below:

parameters.time = 30; %(for S2R+ cells, 20 for S2 cells)
parameters.PixelSize =160; %Pixel Size of instrument in nm
parameters.initialthreshold = 5;
parameters.SNR = 3;
parameters.max_spot_size =4; %Maximum Spot Size [radius in pixel]
parameters.minLength = 5; %defines minimal length of tracks to be kept [frames]
parameters.max_step = 3; %defines maximal distance at which 2 spots are linked in subsequent frames [pixels]
parameters.memory = 0; %this is the number of time steps that a particle can be 'lost' and then recovered again [frames]
parameters.step = 5; %Number of points for calculating single MSD and for fit the single trajectories
parameters.n_fit = 5; %Number of points for fitting the ensemble plot
parameters.lobject = 1.5;
parameters.lnoise = 1;
parameters.pkfnd_sz = 0.5;
parameters.cntrd_sz = 2.5;

To test the validity of our analysis, we used Monte-Carlo simulations to generate data of two dimensional Brownian diffusers with known diffusion coefficients, diffraction-limited size, and the same signal-to-background sampled from a distribution levels determined from our experimental data. These simulated data were analysed using the same track detection and fitting protocol of the real data, an example is which is included at Suppl. Movie 6.

Supplementary Figure 3: Localisation Precision of representative data

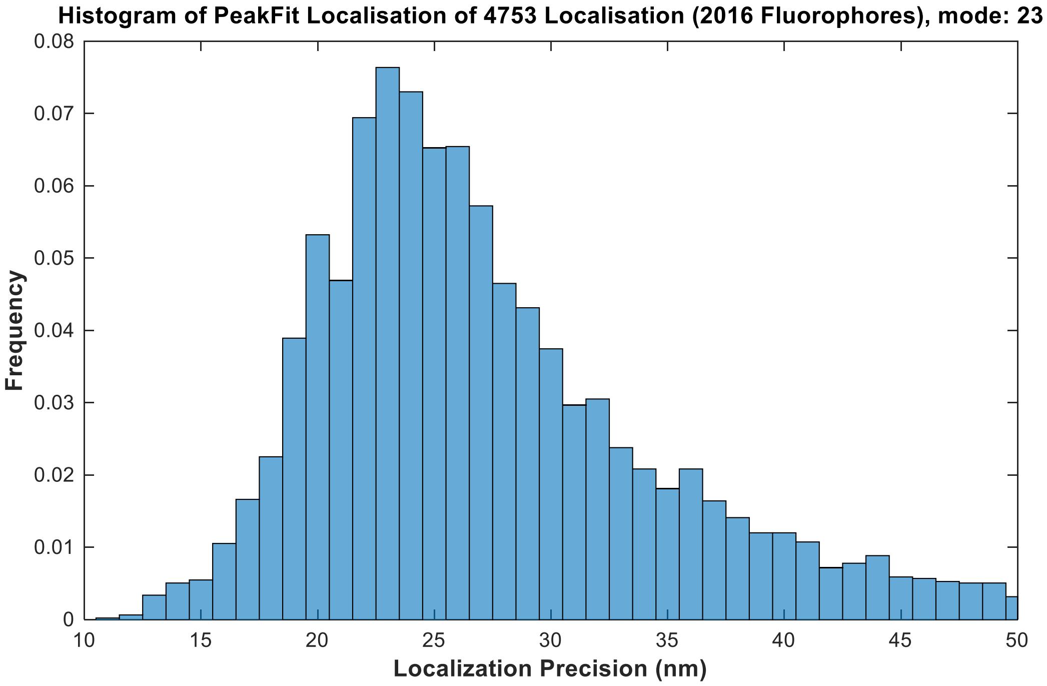

Supplementary Figure 3. Localisation precision was determined using the freely available localization software PeakFit (http://www.sussex.ac.uk/gdsc/intranet/microscopy/imagej/smlm_plugins) from S2 cell diffusion data (4753 Localisation of 2016 Fluorophores).

Simulation of single-particle diffusion

Single-particle diffusion was simulated using the MatLab package ICSMatLab (www.cellmigration.org/resource/imaging/icsmatlab/ICSTutorial.html). This package generates image sequences of Gaussian spots diffusing. Normally distributed signal and background noise is added at to match the quality of the experimental data. The spots are translated by step sizes sampled from a normal distribution based on a specified diffusion coefficient. This code was modified to create Gaussians with subpixel localization. Multiple simulations with different diffusion coefficients were run and added together to generate image sequences representing multiple diffusing populations.

It has been known that MSD is influenced not only by static errors but also by dynamic errors due particle moving while acquisition (3-7). To accommodate for this motion blur we subsampled simulated by a factor of ten. The factor of ten was chosen, so that the MSD during a subsampled frame at the given diffusion coefficient would be less than the localisation precision. The frames were then summed and signal and background values as well as their variances were adjusted to be comparable or slightly poorer than real data (see Suppl. Movie 6).

Simulation (mean +/- StDev):	Background:	1230 +/- 250,	Signal: 1933 +/- 519
Data (mean +/- StDev):	Background:	1128+/- 191,	Signal: 1731 +/- 511

The simulated data gave a mean track length of 8, with a mean SNR over the tracks of 5.8 (real data: mean track length: 12, SNR: 9.2).

Supplementary Figure 4: Freely diffusing Brownian motion of simulated particles

The MSD curve was fitted accommodating static and dynamic errors (3-7) in the form of:

$$< x^2 >= 4Dnt_e + 4\,\sigma^2 - 4/3Dt_e$$

With D being the diffusion coefficient, σ the localization precision of motion blurred particles, n the number of frames and t_e the exposure time.

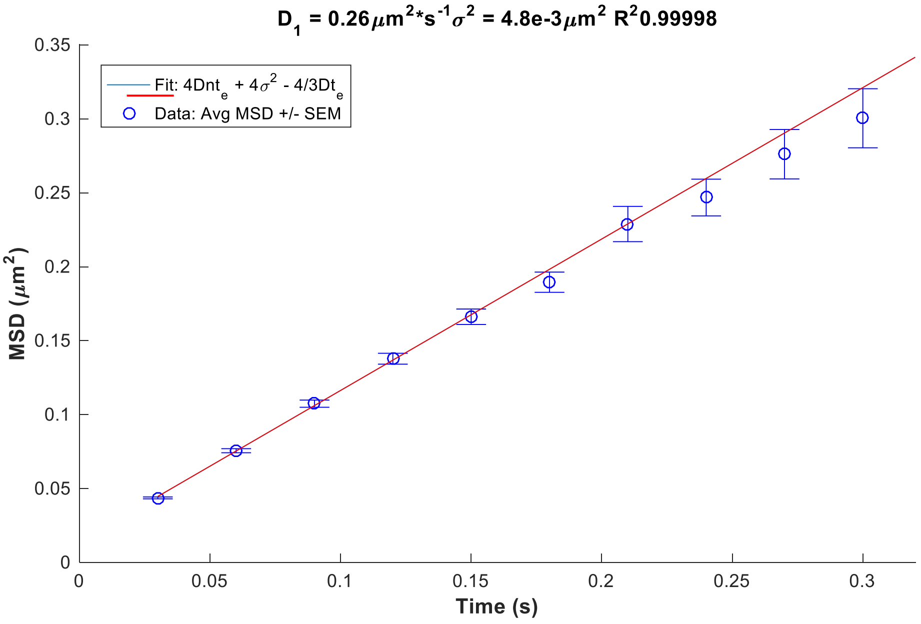

Supplementary Figure 4. MSD Analysis of simulated Data of freely diffusing, motion blurred particles with D = 0.3 $\mu m^2 s^{-1}$, 753 tracks, mean track SNR = 5.8, mean track length 8 at 30 ms exposure and 160 nm pixel size. Plotted is the average MSD curve with SEM as well as the linear fit (red).

Supplementary Figure 5: Wnt3A proteins stay longer associated with receptor expressing S2R+ cells than receptor free S2 cells.

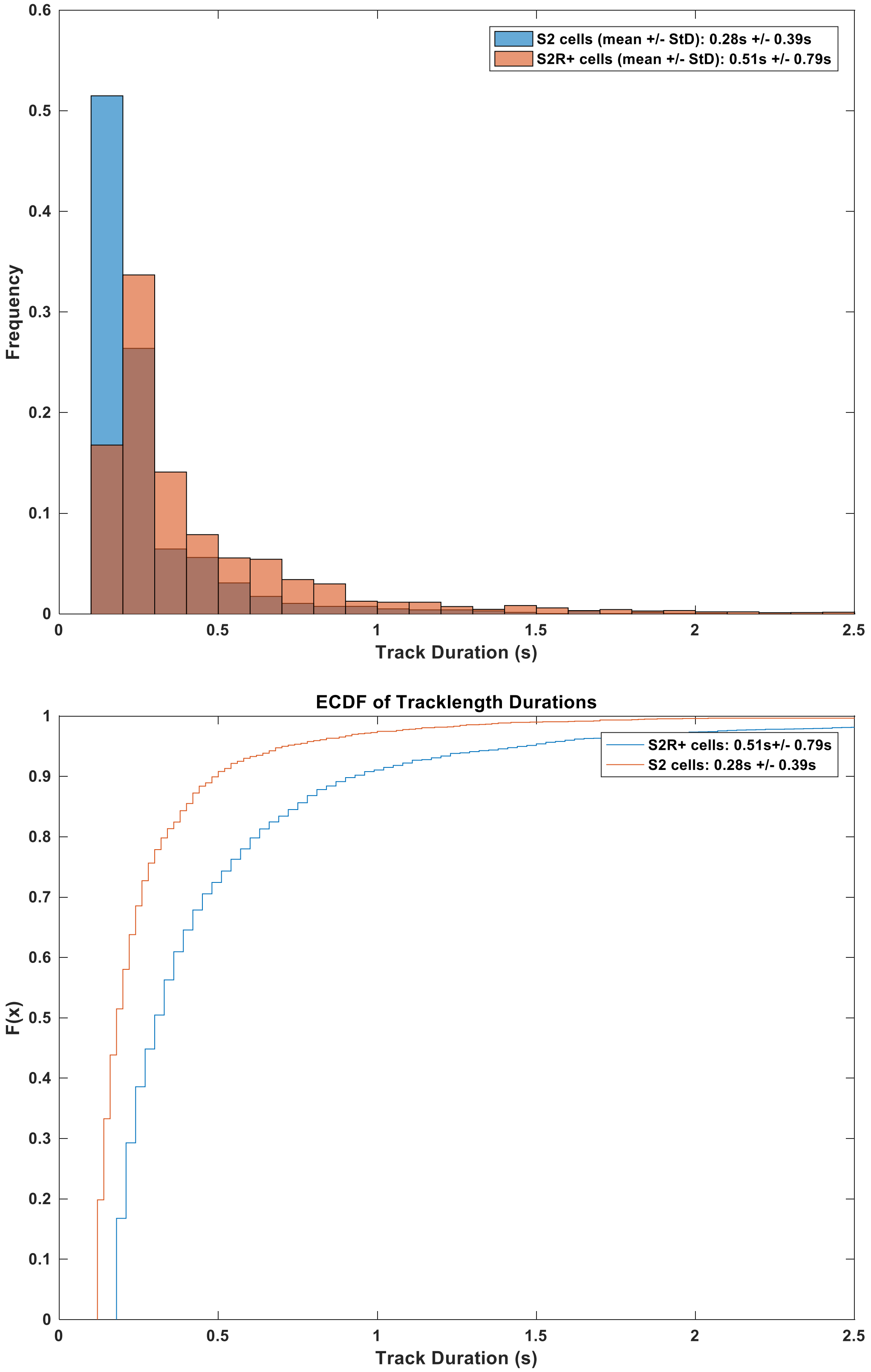

Supplementary Figure 5. Wnt3A Protein stays longer in contact with S2R+ cells than receptor free S2 cells. Shown is the track length distribution of S2 cells (2016 tacks, 5 cells, mean SNR: over track: 9.2) and S2R+ cells (3008 tracks, 8 cells, mean SNR over tracks: 8.7)

Supplementary Figure 6: Continuous replenishment of diffusing Wnt3A proteins on cell membrane.

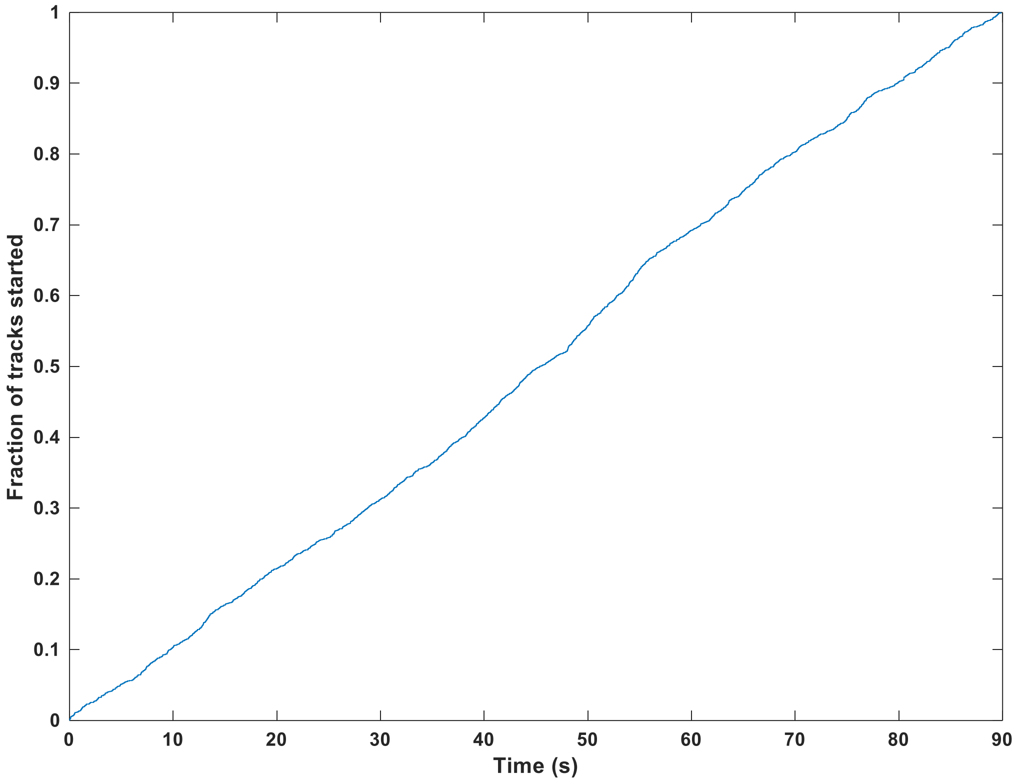

Supplementary Figure 6. Track fraction as a function of time. The rate at which single tracks start is uniform over the acquisition time indicating a constant binding and unbinding of Wnt3A proteins to the membrane. Shown here are the fractions of tracks started in each frame in a S2 cell (2108 tracks).

References

1. PL Weimann, L., Ganzinger, K.A., McColl, J., Irvine, K.L., Davis, S.J., Gay, N.J., Bryant, C.E., Klenerman, D. A Quantitative Comparison of Single-Dye Tracking Analysis Tools Using Monte Carlo Simulations (2013) PLoS ONE, 8 (5), art. no. e64287,

2. Watkins, L.P., Yang, H. Detection of intensity change points in time-resolved single-molecule measurements (2005) Journal of Physical Chemistry B, 109 (1), pp. 617-628.

3. A. J. Berglund, Statistics of camera-based single-particle tracking, Phys. Rev. E 82, 011917 (2010).

4. T. Savin and P. S. Doyle, Static and dynamic errors in particle tracking microrheology, Biophys. J. 88, 623 (2005).

5. X. Michalet and A. J. Berglund, Optimal diffusion coefficient estimation in single-particle tracking, Phys. Rev. E 85, 061916 (2012).

6. X. Michalet, Mean square displacement analysis of singleparticle trajectories with localization error: Brownian motion in an isotropic medium, Phys. Rev. E 82, 041914 (2010).

7. Mikael P. Backlund, Ryan Joyner and W. E. Moerner, Chromosomal locus tracking with proper accounting of static and dynamic errors, Phys. Rev E 91, 062716 (2015).
